# Content validity and reliability of test of gross motor development in Chilean children

**DOI:** 10.1590/S0034-8910.2015049005724

**Published:** 2016-01-14

**Authors:** Marcelo Cano-Cappellacci, Fernanda Aleitte Leyton, Joshua Durán Carreño

**Affiliations:** I Departamento de Kinesiología. Facultad de Medicina. Universidad de Chile. Santiago, RM, Chile; II Escuela de Kinesiología. Facultad de Medicina. Universidad de Chile. Santiago, RM, Chile

**Keywords:** Child, Motor Skills, Reproducibility of Results, Validity of Tests, Validation Studies, Translations

## Abstract

**OBJECTIVE:**

To validate a Spanish version of the Test of Gross Motor Development (TGMD-2) for the Chilean population.

**METHODS:**

Descriptive, transversal, non-experimental validity and reliability study. Four translators, three experts and 92 Chilean children, from five to 10 years, students from a primary school in Santiago, Chile, have participated. The Committee of Experts has carried out translation, back-translation and revision processes to determine the translinguistic equivalence and content validity of the test, using the content validity index in 2013. In addition, a pilot implementation was achieved to determine test reliability in Spanish, by using the intraclass correlation coefficient and Bland-Altman method. We evaluated whether the results presented significant differences by replacing the bat with a racket, using T-test.

**RESULTS:**

We obtained a content validity index higher than 0.80 for language clarity and relevance of the TGMD-2 for children. There were significant differences in the object control subtest when comparing the results with bat and racket. The intraclass correlation coefficient for reliability inter-rater, intra-rater and test-retest reliability was greater than 0.80 in all cases.

**CONCLUSIONS:**

The TGMD-2 has appropriate content validity to be applied in the Chilean population. The reliability of this test is within the appropriate parameters and its use could be recommended in this population after the establishment of normative data, setting a further precedent for the validation in other Latin American countries.

## INTRODUCTION

The early assessment of motor skills and development has gained great importance recently. International evidence points out to positive aspects on mastering mature motor patterns, which will contribute to physical, cognitive and social development.^5^ ^,^ ^6^ ^,^ ^15^ Basic motor skills (BMS) are the foundations that lead to more complex movement sequences. Previously learned skills are refined and combined to be used in the most demanding situations. They contribute to the participation of children, adolescents, and adults in physical activities requiring control of their body in the space, control of antigravity muscles, and precise control of different objects; basic skills for locomotion, stability, and handling, respectively.^6^ The mastering of BMS is correlated with different health benefits, related to body weight status, cardiovascular health, and level of physical activity performed by children.^8^ ^,^ ^11^

Chile is a country with high prevalence of childhood obesity, sedentary lifestyle, and cardiovascular diseases.^a^ Thus, a motor assessment tool is necessary to investigate the relationship between these health variables and the mastering of BMS and its implications. Investigations about BMS have been done in Chile, using tools without the adequate prior validation process.^7^ The international literature describes different types of instruments that allow to assess motor development at different stages of life.^3^ Few Chilean studies on the validation of new tools to the national context exist. On the other hand, instruments created and validated for Chilean children have been used for over 30 years, allowing a follow-up up to five years of age. Also, no national statistics that allow knowing the level of motor development with which children start their school life are available.

One of the most used tools in the international literature is the Test of Gross Motor Development version 2 (TGMD-2), designed by Dale Ulrich, in the United States. This tool aims to identify children with deficits in gross motor development, from three to 10 years of age, evaluating 12 BMS grouped in two subtests: one of locomotor skills and other of object control skills. It is a test focused on the process and on the quality of the movement of a particular BMS. It enables to compare the performance of each individual with pre-established movement quality criteria, or with regulatory results of a representative and statistical sample of the population on which the test is validated.^18^ Normative data is representative of a specific population; thus, the use of the test in other countries is limited to the existence of normative values for this population in particular.^3^ The test has adequate validity and reliability for the USA population, with values of r = 0.88, for test-retest, and 0.98, for inter-rater reliability. However, no intra-rater reliability reports exist.^18^ The TGMD-2 has been validated in countries such as China, Belgium, Brazil, among others, which shows the international interest in this tool.^14^ ^,^ ^19^ ^,^ ^21^ In Chile, progress has been made on this matter, with initiatives throughout the country, in which the test is used in children.^7^ However, a validation process like this has not been developed yet. This study aims to complement those pioneering initiatives, providing the validation of the test in Chile with greater methodological rigor.

It is interesting to investigate the reproducibility of the test in other countries, since it was designed in a specific context. For example, the batting test is considered an important skill for pre-sports development of American boys and girls. However, in Chile and most countries in Latin America, it is not a widely practiced skill.

The purpose of this study was to validate a version in Spanish of the test of gross motor development (TGMD-2) for the Chilean population.

## METHODS

This is a descriptive study, part of the validation process of the gross motor instrument, TGMD-2. The study was conducted in three phases. The first consisted of a process of translation and back-translation to obtain a version of the TGMD-2 with translinguistic equivalence to the original test. The second was a content validation. Finally, the third one was an assessment of the inter-rater and intra-rater reliability of the test in Spanish, in addition to test-retest with the purpose of establishing the time stability of the data.

The TGMD-2 was translated from English into Spanish independently by two translators whose native language is Spanish. Two versions of the test were created, which were contrasted by a bilingual investigator to obtain a preliminary version in Spanish. This preliminary version was subjected to a blind and independent back-translation by other two translators, who did not know the original test, thus obtaining two versions of the instrument in its original language. A committee was made up of all the translators: two with knowledge in the area of gross motor skills, and two others familiar with colloquial phrases in both languages. This committee compared the back-translated versions with the original test and the preliminary version in Spanish, thus solving, unanimously, semantic discrepancies, and generating a second version in Spanish (TGMD-2-CH).

The investigation team decided to evaluate a thirteenth skill based on the controversy surrounding the hitting skill. The bat was replaced by a tennis racket, keeping the evaluation criteria, to know how Chilean children behave in the face of both conditions. Therefore, a modified third version, evaluated in parallel to the TGMD-2-CH, was generated.

For content validation, the procedure described by McGartland was conducted.^12^ We contacted three experts, physical education professors specialized in child motor skills, who worked between the years 2011-2013 in the area of motor skills, and knew the instrument TGMD-2. This committee evaluated the content validity of the Spanish version agreed upon by the translators, using a Likert-type scale developed by the investigation team. Experts expressed their degree of agreement according to the relevance and clarity of the language of the translation of the TGMD-2, on a scale of 1 to 5, justifying their score (1: nothing relevant or unclear; 5: very relevant or very clear). Comments were accepted, thus modifying the test to a level of acceptable content validity (≥ 0.8), for the content validity index.^12^

Once approved, the test was applied to the target population to determine its reliability. Data was collected with the TGMD-2-CH already translated into Spanish and approved by experts. Two raters participated, responsible for applying and then independently rate the videos. One of them was responsible for conducting the intra-rater test, while the other was responsible for the test-retest.

The reliability study was conducted in an urban area in the city of Santiago de Chile. A casual nonprobability sampling was used to select the educational institution. All children between five and 10 years enrolled in the year of 2013 were invited to participate (609 students). Children who refused to participate, or who did not have the consent of their parents (517 children) were excluded from this study. We evaluated 92 children, who were filmed performing the motor skills described in the TGMD-2-CH inside the establishment, during physical education classes, and in the presence of a teacher. Assessments were conducted in groups of three persons according to the test instructions. The recordings were rated by the corresponding rater(s).

To determine the test-retest reliability, 32 children were randomly selected, who were recorded and evaluated for the second time by the rater, two weeks after the first evaluation. To determine the intra-rater agreement, 32 children were randomly selected, and a rater evaluated on two occasions, with an interval of one month, the same video of these children. These periods were determined based on the existing literature and on protocols used in similar studies. These times are considered as appropriate intervals to minimize possible memory bias of the rater, when evaluating again the same child.^1^ ^,^ ^10^ ^,^ ^16^

The data was analyzed with the SPSS Program version 20, using measures of central tendency and frequency distribution. In the statistical analysis, we used: the content validity index (CVI); t-test to compare the results of the test using bat *versus* results using racket; intra-class correlation coefficient (ICC); and the Bland-Altman method for intra-rater, inter-rater and test-retest reliability.^13^ ^,^ ^17^

This study followed the “International Guidelines for Biomedical Research Involving Human Subjects” from the World Health Organization,^4^ with the approval of the Ethics Commission of the Medical School, University of Chile (Process 074/2013). All participants signed an informed consent form.

## RESULTS

We obtained a CVI = 0.88 in the language clarity evaluation of the TGMD-2-CH, and a CVI = 0.83 for the modified TGMD-2. TGMD-2-CH obtained a CVI = 0.90, and the modified test obtained a CVI = 0.84.

The TGMD-2-CH and modified TGMD-2 were applied in 92 children between five and 10 years old, with average age of 7.5 years (SD = 1.6 years), 56 boys and 36 girls. Raw scores for each subtest (Table 1) were calculated.

**Table 1:** Mean of the raw results of the TGMD-2-CH by sex and age. Santiago de Chile, 2013.

Variable	n	%	Average score TGMD2-CH	Standard deviation
Total sample	92	100	65.5	8.6
Locomotor subtest	92	100	34.7	4.7
Object control subtest	92	100	33.1	4.2
Sex				
Female	36	39.1	61.2	9.1
Male	56	60.9	68.2	7.1
Age				
5 years	16	17.4	57.8	10.1*
6 years	15	16.3	65.2	7.7
7 years	13	14.1	64.6	8.2
8 years	17	18.5	68.9	8.8
9 years	23	25.0	68.2	5.9
10 years	8	8.7	65.5	6.4

TGMD-2: Test of Gross Motor Development version 2

* Significant differences (p < 0.05) of the raw score between the 5-year-old age group and other age groups.

The Kolmogorov-Smirnov test indicated a normal distribution for the studied variables, for which the t-test was used to determine if there were significant differences between using a bat or a racket. We did not observe any significant differences (p = 0.059) in assessing the total score for both tests. However, we observed statistically significant differences (p = 0.038) when comparing the results of the object control subtest.

We randomly selected 32 minors (12 girls) to determine the inter-rater concordance. When analyzing the results of both raters, significant differences between the results of both were obtained (p = 0.006), and a CVI = 0.86 for the total score of the TGMD-2-CH, a CVI = 0.87 for the locomotor subtest, and a CVI = 0.88 for the object control subtest (Table 2). We obtained between 0.82 and 0.89 for each of the skills in the locomotor subtest, while, in the object control subtest, we obtained values between 0.80 and 0.92. The modified TGMD-2 obtained CVI = 0.83 in its total score, CVI = 0.85 for the control object subtest, and score of 0.63 in the skill to hit a ball with a racket.

**Table 2:** Results of reliability tests. Santiago de Chile, 2013.

Variable	Age (years)		Sex		Evaluation 1 (score)		Evaluation 2 (score)		Concordance		T-test

	Mean	DE	Female	Male	Mean	DE	Mean	DE	CVI	95%CI	p
Inter-rater (n = 32)	8.2	1.4	12	20							
Total score					69.5	6.3	67.0	7.6	0.86	0.72;0.93	0.006
Locomotor subtest					36.7	3.3	36.1	3.5	0.87	0.73;0.93	0.14
Object control subtest					32.7	5.5	30.9	6.3	0.88	0.77;0.94	0.01
Intra-rater (n = 38)	7.5	1.9	15	23							
Total score					66.9	9.2	66.4	9.8	0.91	0.83;0.95	0.91
Locomotor subtest					35.2	4.9	35.1	5.1	0.92	0.83;0.95	0.92
Object control subtest					31.6	6.6	31.3	7.5	0.86	0.76;0.93	0.86
Retest test (n = 32)	7.2	1.3	12	20							
Total score					61.6	8.4	61.8	8.3	0.88	0.75;0.94	0.88
Locomotor subtest					32.5	5.1	32.4	4.5	0.86	0.71;0.93	0.86
Object control subtest					29.1	5.5	29.4	5.6	0.80	0.59;0.90	0.80

TGMD-2: Test of Gross Motor Development version 2

Results are expressed in mean and standard deviation.

We assessed 38 children randomly (15 girls and 23 boys) to determine the intra-rater concordance. We did not observe any significant differences between the results of both evaluations (p = 0.55). The CVI value for intra-rater concordance of the total score of the GMD-2-CH was 0.91, with CVI = 0.92 for the locomotor subtest and CVI = 0.86 for the object control subtest. For the intra-rater concordance of the modified test, a CVI = 0.93 was obtained for the total score and the object control subtest.

We evaluated 32 children (12 girls and 20 boys) to determine test-retest concordance. We did not obtain any significant differences between the results of both evaluations (p = 0.84). The CVI value for the total score of the TGMD-2-CH was 0.88, while for the modified test was 0.90. In the locomotor subtest, the CVI was 0.86; in the object control subtest with bat was 0.80, while with racket was 0.89.

**Figure 1:**
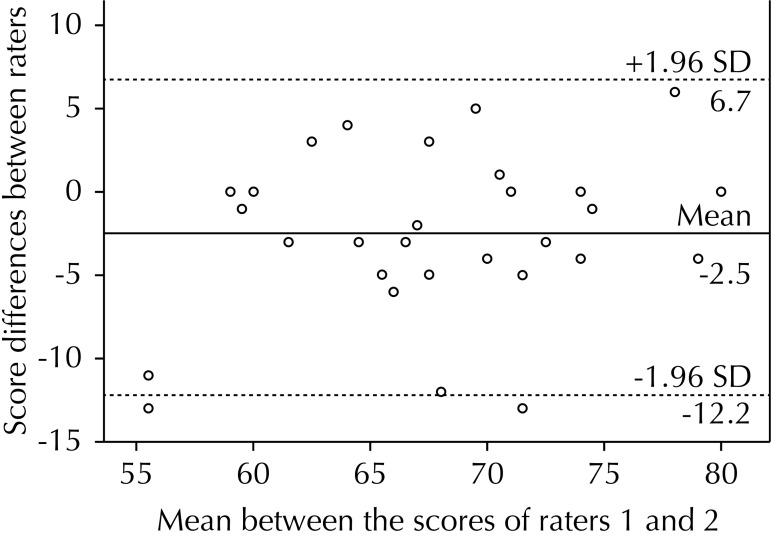
Bland-Altman inter-rater. Comparison of TGMD-2-CH scores obtained by two independent raters. Santiago de Chile, 2013. (N = 32)

**Figure 2:**
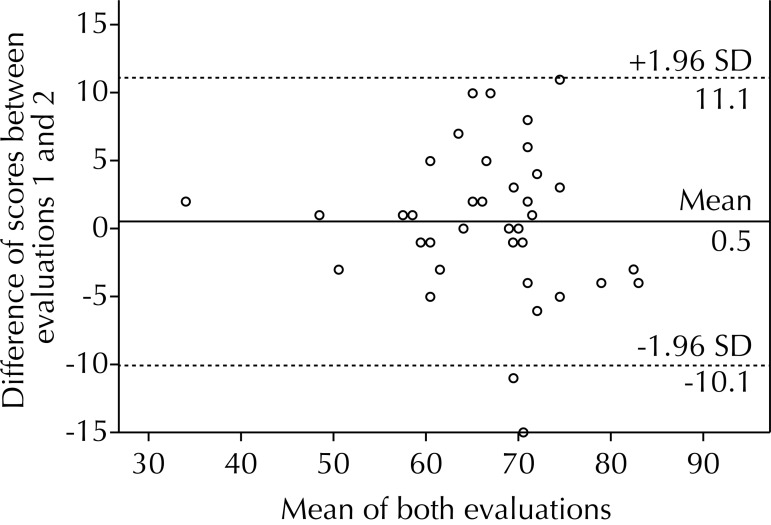
Bland-Altman inter-rater. Comparison of the scores obtained to assess the same video twice. Santiago de Chile, 2013. (N = 38)

**Figure 3:**
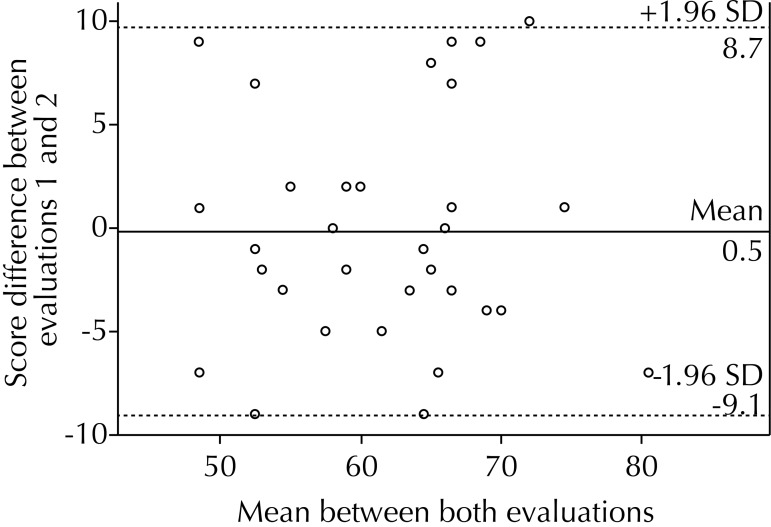
Bland-Altman Test-retest. Comparison of TGMD-2-CH scores achieved by a sample evaluated twice. Santiago de Chile, 2013. (N = 32)

Data was distributed homogeneously in the inter-rater, intra-rater and test-retest concordance, concentrating most of them within two standard deviations (Figures 1, 2 and 3). We have observed more values below the 0 line, being the average of the differences -2.5 points in the inter-rater graph (Figure 1). There was a partially homogenous distribution above and below the 0 line, existing only 1.0% of difference between the two assessments with average of the differences of -0.5 points in the intra-rater graph (Figure 2). We observed a homogeneous distribution above and below the zero line, obtaining a negative average of -0.2 points for the test-retest graph (Figure 3).

TGMD-2: Test of Gross Motor Development version 2

TGMD-2: Test of Gross Motor Development version 2

TGMD-2: Test of Gross Motor Development version 2

## DISCUSSION

In the light of the relations established between the mastery of BMS and factors such as cardiorespiratory status, obesity, long term physical activity, academic performance, among others,^11^ ^,^ ^20^ it is important to have a method for the evaluation of motor skills promoting the investigation and knowledge of this area in the Chilean population. The TGMD-2 has established itself as one of the major evaluation tools, which encourages its validation around the world.^9^ ^,^ ^20^ It is vital to support the use of an evaluation tool, through the knowledge of the reliability and content validity values, with the purpose of obtaining a standardized tool for massive use. Without this support, the obtained results could be inaccurate concerning motor development evaluation, thus causing improper use of resources.^1^

This study generated a version of the TGMD-2 in Spanish, with high content validity index,^12^ based on a clear and appropriate language for the Chilean population. For this purpose, an independent translation process and the formation of a panel were required to solve discrepancies and generate a final version, which diminished the subjectivity bias of each translator.

Our results are similar to those of Valentini^19^ (CV = 0.93 for language clarity, and CV = 0.91 for relevance). However, the values obtained in the modified TGMD-2 are smaller, reaching adequate indexes, but close to the lower limit. The experts rated the skill with racket application as of low relevance for different reasons: the racket weight, or its size, could affect the performance of the children’s motor skill. Although relevant safeguards for the pilot implementation have been taken, selecting a racket weight similar to the bat weight and adequate size for children, effectively replacing the bat with a racket affected the result of the last test criterion: “the bat/racket contacts the ball”. This happens because the racket has more surface area for contact, and therefore, it is easier, in a way that this criterion does not discriminate between children who have greater precision and those who do not. One of the experts pointed out, based on his studies, that although the hitting skill is not usual in the Chilean culture, children have good performance of this motor skill.^7^

The inter-rater, intra-rater and test-retest reliability were found to be high according to the values established by Streiner:^16^ the CVI is higher than 0.8 in all of these, so it has proper concordance. The intra-rater reliability obtained concordance values for the score per subtest and for the total score higher than for the inter-rater reliability in similar studies.^10^ ^,^ ^19^ This relates to the rater’s subjectivity, because although there is clear description to rate whether or not he or she meets certain criteria, the determination of whether his or her performance is or not creditor of the positive rating will always be subject to the rater’s discretion, which may not always be equal to the second rater.

In the graphic representation using the Bland-Altman method for intra-rater concordance (Figure 2), we could inferred that, in the second evaluation, slightly higher scores were assigned, although without significant differences between them (p = 0.55). We obtained a negative average for the total test-retest score (Figure 3), from which we deduce that the second evaluation was slightly lower than the first, but without statistical significance (p = 0.84). There are more values under the 0 line in the graph for the inter-rater concordance (Figure 1), thus indicating that the second rater gave children a higher grade, reaching statistical significance (p = 0.006). There are studies not showing significant differences; however, we observe values close to the significance limits.^10^ However, the magnitude of this difference is less than 5.0% of the total score.

Although the reliability values obtained are lower than those obtained by Ulrich^18^ in the original test in the USA population, he used correlation statistic, and not concordance statistic. The results of this study show an acceptable level of validity and reliability,^16^ thus the TGMD-2-CH could be used in Chilean population without drawbacks. Therefore, we propose it as the tool of choice to determine the level of motor development of Chilean children, due to its wide application as a follow-up and evaluation method,^6^ ^,^ ^18^ and its worldwide use.^9^ ^,^ ^19^ ^,^ ^20^

The limitations of this study were the sample size, and the age group of the sample, since they constitute only a segment of the target population. The question of what happens to Chilean children from three to four years old remain a pending challenge for further investigations. It is also a challenge to establish in the future normative values for Chilean population, to adjust the raw scores according to age, thus completing the validation process of the TGMD-2-CH. We propose to perform test reliability studies in a representative sample of the Chilean population, to obtain definitive results with reliability values greater than those obtained in this study, and similar to those found in the literature.^2^ ^,^ ^19^ We also propose to expand the investigation around the validation of instruments in Chile and Latin America. The use of non-validated instruments involves building knowledge on questionable grounds, which could mean a significant loss of efforts and resources.

## Footnotes

a Organization for Economic Co-operation and Development. Health at a glance 2011: OECD indicators. Paris; 2011 [cited 2015 May 19]. Available from: http://www.oecd.org/els/health-systems/49105858.pdf
