# Supplementary material for: Content validity and reliability of test of gross motor development in Chilean children
Source: Rev Saude Publica. 2016 Jan 14;49:97. doi: 10.1590/S0034-8910.2015049005724 (PMC4760713; doi:10.1590/S0034-8910.2015049005724)
Supplement: Translation

# Translation: Confiabilidad y validez de contenido de test de desarrollo motor grueso en niños chilenos

**Authors:** Marcelo Cano-Cappellacci, Fernanda Aleitte Leyton, Joshua Durán Carreño

**Affiliations:** I Departamento de Kinesiología. Facultad de Medicina. Universidad de Chile. Santiago, RM, Chile; II Escuela de Kinesiología. Facultad de Medicina. Universidad de Chile. Santiago, RM, Chile

**Keywords:** Niño, Destreza Motora, Reproducibilidad de Resultados, Validez de las Pruebas, Estudios de Validación, Traducciones

## Abstract

**OBJETIVO:**

Validar una versión en español de la prueba de desarrollo motor grueso TGMD-2 para la población chilena.

**MÉTODOS:**

Estudio de validez y confiabilidad, no experimental, transversal, de tipo descriptivo. Participaron cuatro traductores, tres expertos y 92 niños chilenos, entre cinco y 10 años, alumnos de un colegio de Santiago de Chile. Se realizaron los procedimientos de traducción, retro-traducción y revisión, por parte del comité de expertos, para determinar la equivalencia trans-lingüística y la validez de contenido del test, usando el índice de validez de contenido en 2013. También se realizó una aplicación piloto para determinar la confiabilidad del test en español, utilizando el coeficiente de correlación intraclase y el método Bland-Altman. Se evaluó si existían diferencias significativas en los resultados, al reemplazar el bate por una raqueta, usando la prueba T-test.

**RESULTADOS:**

Se obtuvo un índice de validez de contenido mayor de 0,8 para pertinencia y claridad del lenguaje del test TGMD-2 para los niños. Hubo diferencias significativas en el subtest de control de objetos, al comparar los resultados con bate y raqueta. El coeficiente de correlación intraclase para la confiabilidad interevaluador, intraevaluador y test re-test, resultó ser mayor de 0,8 en todos los casos.

**CONCLUSIONES:**

El test TGMD-2 presenta adecuada validez de contenido para ser aplicado en la población chilena. La confiabilidad de este test está dentro de los parámetros adecuados y su uso sería recomendado en esta población luego de establecer los datos normativos, sentando un precedente adicional para la validación en otros países latinoamericanos.

## INTRODUCCIÓN

La evaluación temprana del desarrollo y habilidades motrices ha cobrado gran importancia recientemente. Existe evidencia internacional que señala los aspectos positivos sobre los cuales repercute dominar patrones motores maduros que contribuirán al desarrollo físico, cognitivo y social.^5^ ^,^ ^6^ ^,^ ^15^ Las habilidades motoras básicas (HMB) son los cimientos que conducen a secuencias de movimiento más complejas. Las habilidades previamente aprendidas son perfeccionadas y combinadas para ser usadas en situaciones cada vez más demandantes. Contribuyen en la participación de niños, adolescentes y adultos en actividades físicas que requieran control de su cuerpo en el espacio, control de la musculatura anti-gravitatoria y control preciso de diferentes objetos; habilidades esenciales para la locomoción, la estabilidad y la manipulación, respectivamente.^6^ El dominio de las HMB se correlaciona con diferentes beneficios en salud, relacionados con el estado del peso corporal, la salud cardiovascular y el nivel de actividad física realizada por los niños.^8^ ^,^ ^11^

Chile es un país con alta prevalencia de obesidad infantil, sedentarismo y enfermedades cardiovasculares.^a^ Así, es importante disponer de una herramienta de evaluación motora para indagar acerca de la relación entre estas variables de salud, con el dominio HMB y sus implicancias. En Chile se han realizado investigaciones en torno a las HMB, utilizando herramientas sin el adecuado proceso de validación previo.^7^ La literatura internacional describe diversos tipos de instrumentos que permiten evaluar el desarrollo motor en las distintas etapas de la vida.^3^ Existen pocos estudios chilenos en torno a la validación de nuevas herramientas para el contexto nacional. Por el contrario, se utilizan instrumentos creados y validados para niños chilenos, desde hace más de 30 años, que permiten un seguimiento hasta los cinco años. Tampoco existen estadísticas nacionales que permitan conocer el nivel de desarrollo motor con el cual los niños inician su periodo escolar.

Una de las herramientas más utilizadas en la literatura internacional es el *Test of Gross Motor Development* versión 2 (TGMD-2), diseñado por Dale Ulrich, en Estados Unidos. Esta herramienta busca identificar a los niños con déficit en el desarrollo motor grueso, entre los tres y 10 años, evaluando 12 HMB agrupadas en dos subtest: uno de habilidades locomotoras, y otro de habilidades de control de objetos. Es un test enfocado en el proceso y en la calidad del movimiento de una determinada HMB. Permite comparar el desempeño de cada individuo con criterios pre-establecidos, de calidad de movimiento, o con los resultados normativos de una muestra estadística y representativa de la población en la que el test está validado.^18^ Los datos normativos son representativos de una población específica; así, el uso del test en otros países está limitado a la existencia de valores normativos para esa población en particular.^3^ El test posee adecuada validez y confiabilidad para la población estadounidense, con valores de r = 0,88, para la prueba test re-test, y de 0,98, para la confiabilidad inter-evaluador. Sin embargo, no existen reportes de confiabilidad intraevaluador.^18^ El TGMD-2 ha sido validado en países como China, Bélgica, Brasil, entre otros, lo que muestra el interés internacional por esta herramienta.^14^ ^,^ ^19^ ^,^ ^21^ En Chile, se ha avanzado al respecto, con iniciativas a lo largo del país, en las cuales se utiliza el test en niños;^7^ no obstante, aún no se ha desarrollado un proceso de validación como tal. Este estudio pretende complementar aquellas iniciativas pioneras, aportando con mayor rigurosidad metodológica a la validación del test en Chile.

Resulta interesante indagar en la reproductibilidad del test en otros países, ya que este fue diseñado en un contexto determinado. Por ejemplo, la prueba de batear es considerada una habilidad importante para el desarrollo pre-deportivo de los niños y niñas estadounidenses. No obstante, en Chile y en la mayoría de los países en Latinoamérica, esa no es una habilidad ampliamente practicada.

El objetivo de este estudio fue validar una versión en español de la prueba de desarrollo motor grueso TGMD-2 para la población chilena.

## MÉTODOS

Estudio descriptivo que constituye parte del proceso de validación del instrumento de motricidad gruesa TGMD-2. El estudio se desarrolló en tres fases. La primera constó de un proceso de traducción y retro-traducción para obtener una versión del TGMD-2 con equivalencia translingüística con el test original. La segunda, de una validación de contenido; y la tercera, de una evaluación de la confiabilidad interevaluador e intraevaluador del test en español, además del test re-test, con el objeto de establecer la estabilidad temporal de los datos.

El TGMD-2 fue traducido del inglés al español independientemente por dos traductores, cuya lengua nativa es el español. Fueron generadas dos versiones del test, las cuales fueron contrastadas junto a un investigador bilingüe, para obtener una versión preliminar en español. Esta versión preliminar fue sometida a una retro-traducción ciega e independiente por otros dos traductores, quienes no conocían el test original, obteniendo nuevamente dos versiones del instrumento en su lenguaje original. Se conformó un comité con todos los traductores: dos con conocimiento en el área de la motricidad gruesa, y otros dos familiarizados con frases coloquiales de ambos idiomas. Este comité contrastó las versiones retro-traducidas con el test original y con la versión preliminar en español, resolviendo, por unanimidad, discrepancias semánticas, y generando una segunda versión en español (TGMD-2-CH).

El equipo de investigación decidió evaluar una decimotercera habilidad, con base en la controversia en torno a la habilidad de batear. Se reemplazó el bate por una raqueta de tenis, manteniendo los criterios de evaluación, para conocer cómo se comportan los niños chilenos frente a ambas condiciones. Por tanto, se generó una tercera versión, modificada, que se evaluó paralelamente al TGMD-2-CH.

Para la validación de contenido, se llevó a cabo el procedimiento descrito por McGartland.^12^ Se contactó a tres expertos, profesores de educación física especialistas en motricidad infantil, quienes trabajaron entre los años 2011 y 2013 en el área de la motricidad, y conocían el instrumento TGMD-2. Este comité evaluó la validez de contenido de la versión en español consensuada por los traductores, utilizando una escala tipo Likert confeccionada por el equipo de investigación. Los expertos expresaron su grado de acuerdo con la pertinencia y claridad del lenguaje de la traducción del test TGMD-2, en una escala de 1 a 5, justificando su puntuación (1: nada pertinente o nada claro; 5: muy pertinente o muy claro). Las observaciones fueron acogidas, modificando el test hasta alcanzar un nivel de validez de contenido aceptable (≥ 0,8), para el índice de validez de contenido.^12^

Una vez aprobado, el test fue aplicado en la población objetivo para determinar su confiabilidad. Los datos fueron recolectados con el test TGMD-2-CH ya traducido al español y aprobado por los expertos. En ella, participaron dos evaluadores, quienes se encargaron de aplicar y luego puntuar independientemente los videos. Uno de ellos se encargó de realizar la prueba de intraevaluador, mientras que el otro, de los test re-test.

El estudio de la confiabilidad fue realizado en un sector urbano en la ciudad de Santiago de Chile. Se utilizó un muestreo no probabilístico de tipo causal para seleccionar la institución educativa. Todos los niños entre cinco y 10 años matriculados durante el año 2013 fueron invitados a participar (609 alumnos). Fueron excluidos de este estudio los niños que se negaron a participar, o que no tenían el consentimiento de sus apoderados (517 niños). Fueron evaluados 92 niños, quienes fueron filmados realizando las habilidades motoras descritas en el TGMD-2-CH al interior del establecimiento, durante las clases de educación física, y en presencia de un profesor. Las evaluaciones se realizaron en grupos de tres personas, conforme a las indicaciones del test. Las grabaciones fueron calificadas por el o los evaluadores correspondientes.

Para determinar la confiabilidad test re-test, 32 niños fueron seleccionados al azar, los cuales fueron grabados y evaluados por segunda vez por el evaluador, dos semanas después de la primera evaluación. Para determinar la concordancia intraevaluador, 32 niños fueron seleccionados al azar, y un evaluador calificó en dos oportunidades, con intervalo de un mes, el mismo video de estos niños. Estos periodos fueron determinados con base en la literatura existente y en los protocolos utilizados en estudios similares. Se consideran estos tiempos como intervalos apropiados para minimizar eventual sesgo de memoria del examinador, al evaluar nuevamente al mismo niño.^1^ ^,^ ^10^ ^,^ ^16^

Los datos fueron analizados con el Programa SPSS versión 20, mediante medidas de tendencia central y distribución de frecuencias. En el análisis estadístico, se utilizó: el índice de validez de contenido (CVI); el t-test para comparar los resultados del test usando bate *versus* los resultados usando raqueta; el coeficiente de correlación intra-clase (ICC); y el método Bland-Altman para la confiabilidad intraevaluador, interevaluador y test re-test.^13^ ^,^ ^17^

Este estudio siguió las “Pautas Éticas Internacionales para la Investigación y Experimentación Biomédica en Seres Humanos” de la Organización Mundial de la Salud,^4^ y contó con la aprobación del Comité de Ética de la Facultad de Medicina de la Universidad de Chile (Proceso 074/2013). Los participantes firmaron el proceso de consentimiento informado.

## RESULTADOS

Se obtuvo un CVI = 0,88 en la evaluación de la claridad del lenguaje del test TGMD-2-CH, y un CVI = 0,83 para el TGMD-2 modificado. El TGMD-2-CH obtuvo un CVI = 0,90, y el test modificado obtuvo un CVI = 0,84.

El TGMD-2-CH y TGMD-2 modificado fueron aplicados en 92 niños entre 5 y 10 años, con media de edad de 7,5 años (DE = 1,6 años), 56 hombres y 36 mujeres. Se calcularon los puntajes brutos de cada subtest (Tabla 1).

**Tabla 1:** Media de los resultados brutos del TGMD-2-CH por sexo y edad. Santiago de Chile, 2013.

Variable	n	%	Media puntaje TGMD2-CH	Desviación estándar
Muestra total	92	100	65,5	8,6
Sub-test locomotor	92	100	34,7	4,7
Sub-test control objetos	92	100	33,1	4,2
Sexo				
Mujeres	36	39,1	61,2	9,1
Hombres	56	60,9	68,2	7,1
Edad				
5 años	16	17,4	57,8	10,1*
6 años	15	16,3	65,2	7,7
7 años	13	14,1	64,6	8,2
8 años	17	18,5	68,9	8,8
9 años	23	25,0	68,2	5,9
10 años	8	8,7	65,5	6,4

TGMD-2: Test of Gross Motor Development versión 2

* Diferencias significativas (p < 0,05) del puntaje bruto entre el grupo de 5 años y los demás grupos etarios.

La prueba de Kolmogorov-Smirnov indicó una distribución normal para las variables estudiadas, por lo cual se usó la prueba T para determinar si existían diferencias significativas entre usar un bate o una raqueta. No se observaron diferencias significativas (p = 0,059) al evaluar el puntaje total de ambas pruebas, No obstante, se observaron diferencias estadísticamente significativas (p = 0,038) al comparar los resultados del sub-test de control de objetos.

Fueron seleccionados 32 menores (12 niñas) al azar para determinar la concordancia inter-evaluador. Al analizar los resultados de ambos evaluadores, fueron obtenidas diferencias significativas entre los resultados de ambos (p = 0,006), y un ICC = 0,86 para el puntaje total del TGMD-2-CH, un ICC = 0,87 para el subtest locomotor, y un ICC = 0,88 para el sub-test de control de objetos (Tabla 2). Fueron alcanzados valores entre 0,82 y 0,89 para cada una de las habilidades en el sub-test locomotor, mientras que en el sub-test control de objetos fueron alcanzados valores entre 0,80 y 0,92. El TGMD-2 modificado obtuvo ICC = 0,83 en su puntaje total, ICC = 0,85 para el sub-test de control de objetos, y puntaje de 0,63 en la habilidad golpear una pelota con raqueta.

**Tabla 2:** Resultados de las pruebas de confiabilidad. Santiago de Chile, 2013.

Variable	Edad (años)		Sexo		Evaluación 1 (puntaje)		Evaluación 2 (puntaje)		Concordancia		T test

	Media	DE	Mujeres	Hombres	Media	DE	Media	DE	ICC	IC95%	p
Interevaluador (n = 32)	8,2	1,4	12	20							
Puntaje total					69,5	6,3	67,0	7,6	0,86	0,72;0,93	0,006
S. Locomotor					36,7	3,3	36,1	3,5	0,87	0,73;0,93	0,14
S. Control de objetos					32,7	5,5	30,9	6,3	0,88	0,77;0,94	0,01
Intraevaluador (n = 38)	7,5	1,9	15	23							
Puntaje total					66,9	9,2	66,4	9,8	0,91	0,83;0,95	0,91
S. Locomotor					35,2	4,9	35,1	5,1	0,92	0,83;0,95	0,92
S. Control de objetos					31,6	6,6	31,3	7,5	0,86	0,76;0,93	0,86
Test re-test (n = 32)	7,2	1,3	12	20							
Puntaje total					61,6	8,4	61,8	8,3	0,88	0,75;0,94	0,88
S. Locomotor					32,5	5,1	32,4	4,5	0,86	0,71;0,93	0,86
S. Control de objetos					29,1	5,5	29,4	5,6	0,80	0,59;0,90	0,80

TGMD-2: Test of Gross Motor Development versión 2

Resultados se expresan en media y desviación estándar.

Fueron evaluados 38 niños al azar (15 niñas y 23 niños) a fin de determinar la concordancia intraevaluador. No fueron obtenidas diferencias significativas entre el resultado de ambas evaluaciones (p = 0,55). El valor del ICC para concordancia intraevaluador del puntaje total del TGMD-2-CH fue de 0,91, con ICC = 0,92 para el sub-test locomotor e ICC = 0,86 para el sub-test de control de objetos. Para la concordancia intraevaluador del test modificado, fue obtenido un ICC = 0,93 para el puntaje total y para el sub-test de control de objetos.

Fueron evaluados 32 niños (12 niñas y 20 niños) para determinar la concordancia test re-test. No fueron obtenidas diferencias significativas entre los resultados de ambas evaluaciones (p = 0,84). El valor del ICC para el puntaje total del TGMD-2-CH fue de 0,88, mientras que para el test modificado fue de 0,90. En el sub-test locomotor, el ICC fue 0,86; en el sub-test de control de objetos con bate fue 0,80, mientras que con raqueta fue 0,89.

Existió una distribución homogénea de los datos en la concordancia interevaluador, intraevaluador y test re-test, concentrando la mayoría de ellos dentro de las dos desviaciones estándar (Figuras 1, 2 y 3). Se observan más valores bajo la línea del 0, siendo el promedio de las diferencias -2,5 puntos en el gráfico inter-evaluador (Figura 1). Existió una distribución parcialmente homogénea sobre y bajo la línea del 0, existiendo sólo un 1,0% de diferencia entre las dos evaluaciones con promedio de las diferencias de -0,5 puntos en el gráfico intra-evaluador (Figura 2). Se observó una distribución homogénea sobre y bajo la línea del cero, obteniendo un promedio negativo de -0,2 puntos para el gráfico test re-test (Figura 3).

**Figura 1:** Bland-Altman inter-evaluador. Comparación de los puntajes de TGMD-2-CH obtenidos por dos evaluadores independientes. Santiago de Chile, 2013. (N = 32)

TGMD-2: Test of Gross Motor Development versión 2

**Figura 2:** Bland-Altman inter-evaluador. Comparación de los puntajes obtenidos al evaluar en dos oportunidades un mismo video. Santiago de Chile, 2013. (N = 38)

TGMD-2: Test of Gross Motor Development versión 2

**Figura 3:** Bland-Altman Test re-test. Comparación de los puntajes TGMD-2-CH alcanzados por una muestra evaluada dos veces. Santiago de Chile, 2013. (N = 32)

TGMD-2: Test of Gross Motor Development versión 2

## DISCUSIÓN

A la luz de las relaciones establecidas entre el dominio de las HMB y factores como el estado cardio-respiratorio, obesidad, actividad física a largo plazo, y rendimiento académico, entre otros,^11^ ^,^ ^20^ se torna importante contar con un método de evaluación de habilidades motrices que fomente la investigación y el conocimiento de esta área en la población chilena. El TGMD-2 se ha posicionado como una de las principales herramientas de evaluación, lo cual incentiva su validación alrededor del mundo.^9^ ^,^ ^20^ Resulta de vital importancia respaldar el uso de una herramienta de evaluación, por medio del conocimiento de los valores de confiabilidad y validez de contenido, con el fin de obtener una herramienta estandarizada para uso masivo. Sin este sustento, se podrían obtener resultados imprecisos en lo relacionado a la evaluación del desarrollo motor, con lo cual se podría incurrir en una mala utilización de recursos.^1^

Este estudio generó una versión del test TGMD-2 en español, con alto índice de validez de contenido,^12^ con base en un lenguaje claro y pertinente para la población chilena. Para esto, fue necesario un proceso de traducción independiente, y la conformación de un panel para resolver discrepancias y para generar una versión final, que disminuyera el sesgo de subjetividad propia de cada traductor.

Nuestros resultados son similares con los de Valentini^19^ (CV = 0,93 para claridad del lenguaje, y 0,91 para pertinencia). No obstante, los valores obtenidos en el TGMD-2 modificado son menores, alcanzando índices adecuados, pero cercanos al límite inferior. Los expertos calificaron con baja pertinencia la aplicación de la habilidad con raqueta por varias razones: el peso de la raqueta, o el tamaño de esta, podría afectar el desempeño del gesto motor de los niños. A pesar de que se tomaron los resguardos pertinentes para la aplicación piloto, seleccionando una raqueta de peso similar al del bate, y de tamaño adecuado para niños, efectivamente el cambiar el bate por la raqueta afectó el resultado del último criterio de la prueba: “el bate/la raqueta contacta la pelota”. Esto, porque la raqueta posee mayor área de superficie para el contacto, y por tanto, resulta más fácil, provocando que este criterio no discrimine entre los niños que poseen mayor precisión de los que no la tienen. Uno de los expertos señaló, con base en sus estudios, que pese a que la habilidad de batear no es usual en la cultura chilena, los niños poseen buen desempeño en este gesto motor.^7^

La confiabilidad interevaluador, intraevaluador y test re-test, resultaron ser altas de acuerdo a los valores establecidos por Streiner:^16^ el ICC es mayor a 0,8 en todos estos, por lo cual posee apropiada concordancia. La confianza intra-evaluador obtuvo valores mayores de concordancia para el puntaje por subtest y para el puntaje total, que para la confianza inter-evaluador, al igual que estudios similares.^10^ ^,^ ^19^ Esto guarda relación con la subjetividad del evaluador, pues aunque exista descripción clara para calificar si cumple o no cierto criterio, la determinación de si su desempeño es o no acreedor de la calificación positiva, siempre estará sujeta al criterio del evaluador, que no siempre será igual al del segundo evaluador.

En la representación gráfica utilizando el método Bland-Altman para concordancia intra-evaluador (Figura 2), se puede inferir que en la segunda evaluación se asignaron puntajes levemente mayores, aunque sin diferencias significativas entre ellas (p = 0,55). Se obtuvo un promedio negativo para el puntaje total test re-test (Figura 3), de lo cual se deduce que la segunda evaluación fue levemente menor que la primera, aunque sin significancia estadística (p = 0,84). Existen más valores bajo la línea del 0 en el gráfico para la concordancia inter-evaluador (Figura 1), con lo se infiere que el segundo evaluador puntuó con mayor calificación a los niños, alcanzando significancia estadística (p = 0,006). Sin embargo, la magnitud de esta diferencia es menor al 5,0% del puntaje total. Existen estudios que no presentan diferencias significativas; no obstante, se observan valores cercanos a los límites de significancia.^10^

A pesar de que los valores de confiabilidad obtenidos son menores a los obtenidos por Ulrich^18^ en el test original en población estadounidense, él utilizó estadístico de correlación, y no de concordancia. Los resultados de este estudio muestran un nivel de validez y confiabilidad aceptable,^16^ por lo cual el TGMD-2-CH podría ser utilizado en población chilena sin inconvenientes. Por lo tanto, se propone como la herramienta de elección para determinar el nivel de desarrollo motriz de los niños chilenos, por su amplia aplicación como método de evaluación y seguimiento,^6^ ^,^ ^18^ y por su uso generalizado a nivel internacional.^9^ ^,^ ^19^ ^,^ ^20^

Las limitaciones de este estudio fueron el tamaño muestral, y el rango etario de la muestra utilizada, ya que constituyen solo un segmento de la población objetivo. Queda la interrogante de lo que sucede con los niños chilenos de tres a cuatro años, desafío pendiente para futuras investigaciones. Es también un desafío, establecer en el futuro valores normativos para la población chilena, con el objetivo de ajustar los puntajes brutos según edad, y así concluir el proceso de validación del TGMD-2-CH. Se propone realizar estudios de confiabilidad del test en una muestra representativa de la población chilena, para obtener resultados definitivos, con valores de confiabilidad mayores a los obtenidos en la presente investigación, y similares a los que se han encontrado en la literatura.^2^ ^,^ ^19^ Se propone también ampliar la investigación en torno a la validación de instrumentos en Chile y Latinoamérica. El uso de instrumentos no validados implica construir conocimiento sobre bases cuestionables, lo cual podría significar una pérdida importante de esfuerzos y recursos.

## Footnotes

a Organization for Economic Co-operation and Development. Health at a glance 2011: OECD indicators. Paris; 2011 [citado 2015 mai 19]. Disponible en: http://www.oecd.org/els/health-systems/49105858.pdf
